# Precipitation of string-shaped morphologies consisting of aligned α phase in a metastable β titanium alloy

**DOI:** 10.1038/s41598-018-20386-1

**Published:** 2018-02-01

**Authors:** Hongyi Zhan, Anna Ceguerra, Gui Wang, Julie Cairney, Matthew Dargusch

**Affiliations:** 1 Centre for Advanced Materials Processing and Manufacture, School of Mechanical and Mining Engineering, The University of Queensland, St Lucia, Queensland, 4072 Australia; 2 Australian Centre for Microscopy and Microanalysis and School of Aerospace, Mechanical & Mechatronic Engineering, The University of Sydney, Sydney, NSW 2006 Australia; 3 Australian Institute for Nanoscale Science and Technology, The University of Sydney, Sydney, NSW 2006 Australia

## Abstract

String-shaped morphologies consisting of preferentially aligned lath-shaped α precipitates were observed in the metastable β Ti-6Cr-5Mo-5V-4Al alloy after deformations at high strain rates and elevated temperatures. The morphology and 3-dimentional arrangement of this feature have been elaborated based on the characterizations via a combination of transmission electron microscopy, transmission kikuchi diffraction and atom probe tomography. The 2D projected morphology of the coalescent α laths observed in the etched samples by SEM depends on the metallographic section. All the microstructural observations indicate that dislocation structures are most likely the nucleation sites for the aligned α laths. In addition, an appropriate testing temperature, which can ensure a relatively high diffusion rate of solutes without inducing strong recovery of dislocation structures, is necessary for the occurrence of the string-shaped morphologies.

## Introduction

Beta titanium alloys have been developed for the fabrications of large-section structural components in the aerospace industry due to their high hardenability and improved forgeability^1–4^. It has been widely accepted that the mechanical properties of β titanium alloys greatly depend on the volume fraction, size, spatial distribution and morphology of α precipitates formed during thermo-mechanical processing and/or ageing heat treatment. Therefore, various mechanisms for intragranular α precipitation in β titanium alloys have been proposed and investigated in the past decades, such as the pseudo-spinodal mechanism^5^, the ω-assisted mechanisms^6–9^ and the dislocation-assisted mechanisms^10–13^. The characteristics of dislocation-assisted α precipitation is that one or few specific variants will dominate among all the 12 crystallographically equivalent Burgers variants of α phase with respect to β matrix^14^.

In the past years, the influence of dislocation-assisted α precipitation on the mechanical properties of titanium alloys have been studied. In the research on α/β titanium alloys produced by thermo-mechanical processing, it is reported that the formation of a large number of α precipitates with their c-axes aligned along a specific direction under the influence of dislocations will deteriorate the fatigue resistance of the alloy^15^. Song *et al.*^13^ proposed a two-step ageing heat treatment method for the pre-strained Ti-10Mo-8V-1Fe-3.5Al wt. % (TB3) alloy. It has been proved that the pre-strained and aged samples, in which paralleled α plates of a favoured variant were observed precipitating around dislocations, can attain a higher strength than the conventionally aged samples without sacrificing the ductility. Song *et al.*^16^ also tried ageing the TB3 alloy under a compressive stressed condition for several hours. It is reported that the stress-aged samples exhibited a larger tensile elongation than the conventionally aged samples while staying at a similar strength level. To take fully advantages of this defect-assisted precipitation behaviour, an in-depth understanding of the underlying mechanism is necessary. A large number of relevant investigations based on theoretical calculations^12,17^ and phase-field modelling^14,18^ have been made to elaborate the influence of pre-existing dislocation structures in β matrix on the variant selection of α precipitation in titanium alloys. Among them, Qiu *et al.*^14^ simulated 3-dimentional (3D) morphological patterns for α variants formed on different dislocation configurations on the {112}_β_ slip plane by applying phase-field simulation. However, microstructural characterizations on the dislocation-assisted α precipitates in the previous publications are limited to 2-D projected observations^10,16^ and their spatial distribution have not been reported yet.

The Ti-6Cr-5Mo-5V-4Al wt.% (Ti6554) alloy, a newly-developed metastable beta alloy, can be engineered to attain an excellent combination of fracture toughness and strength^19,20^. Our previous work^21^ applied Split Hopkinson Pressure Bar (SHPB) testing on cylindrical Ti6554 specimens over strain rates from 1000 s^−1^ to 10000 s^−1^ and temperatures from 293 K to 1173 K. A large number of string-shaped morphologies were observed on the cross-section of cylindrical Ti6554 specimens after compressive deformations at 4000 s^−1^ and 10000 s^−1^ when the testing temperature was raised to 873 K. The present paper is focused on the identification and characterization of the observed string-shaped morphologies by a combination of Transmission Electron Microscopy (TEM), Transmission Kikuchi Diffraction (TKD) and 3D Atom Probe Tomography (APT). Specific emphasis has been placed on explaining the necessary conditions required by the formation of string-shaped morphologies.

## Results

### The initial microstructure

**Figure 1:**
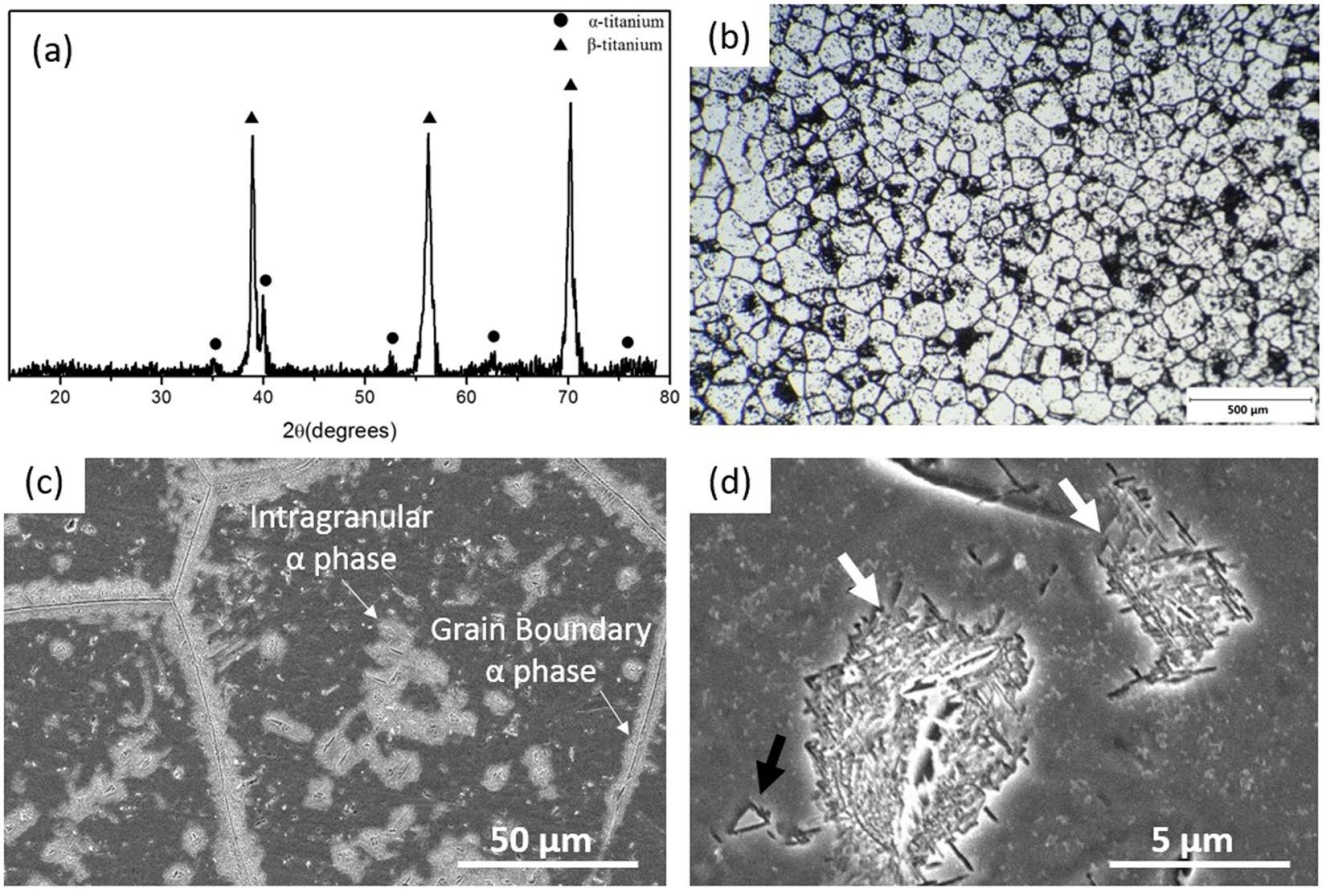
(**a**) XRD spectrum, (**b**) optical image and (**c**) SEM image of initial microstructures of undeformed Ti6554 alloy (solution and ageing heat treated); (**d**) typical intragranular α precipitates (white arrows indicate Widmanst$\ddot{{\rm{a}}}$tten morphology and black arrow indicates tent-like morphology).

According to the XRD spectrum shown in Fig. 1a, the microstructures of undeformed Ti6554 specimens mainly consist of titanium α and β phases. An optical image of the initial microstructure is shown in Fig. 1b and the β grain size is in the range of ~50–120 μm. Figure 1c shows β matrix with α precipitates clustering inside grains and nucleating along grain boundaries. At a higher magnification (Fig. 1d), the morphologies of intragranular α phase are mainly in a mesh consisting of fine Widmanstätten plates or in a tent-like configuration. Similar α morphologies have been reported in the Ti-5Al-5Mo-5V-3Cr (Ti5553) alloy aged at 673 K or higher temperatures^22^. According to the heat treatment studies on the Ti6554 alloy conducted by Li *et al.*^23^, it is apparent that the volume fraction of α precipitates in the initial microstructure of our Ti6554 samples is far away from equilibrium state.

### Characterization of string-shaped morphologies

**Figure 2:**
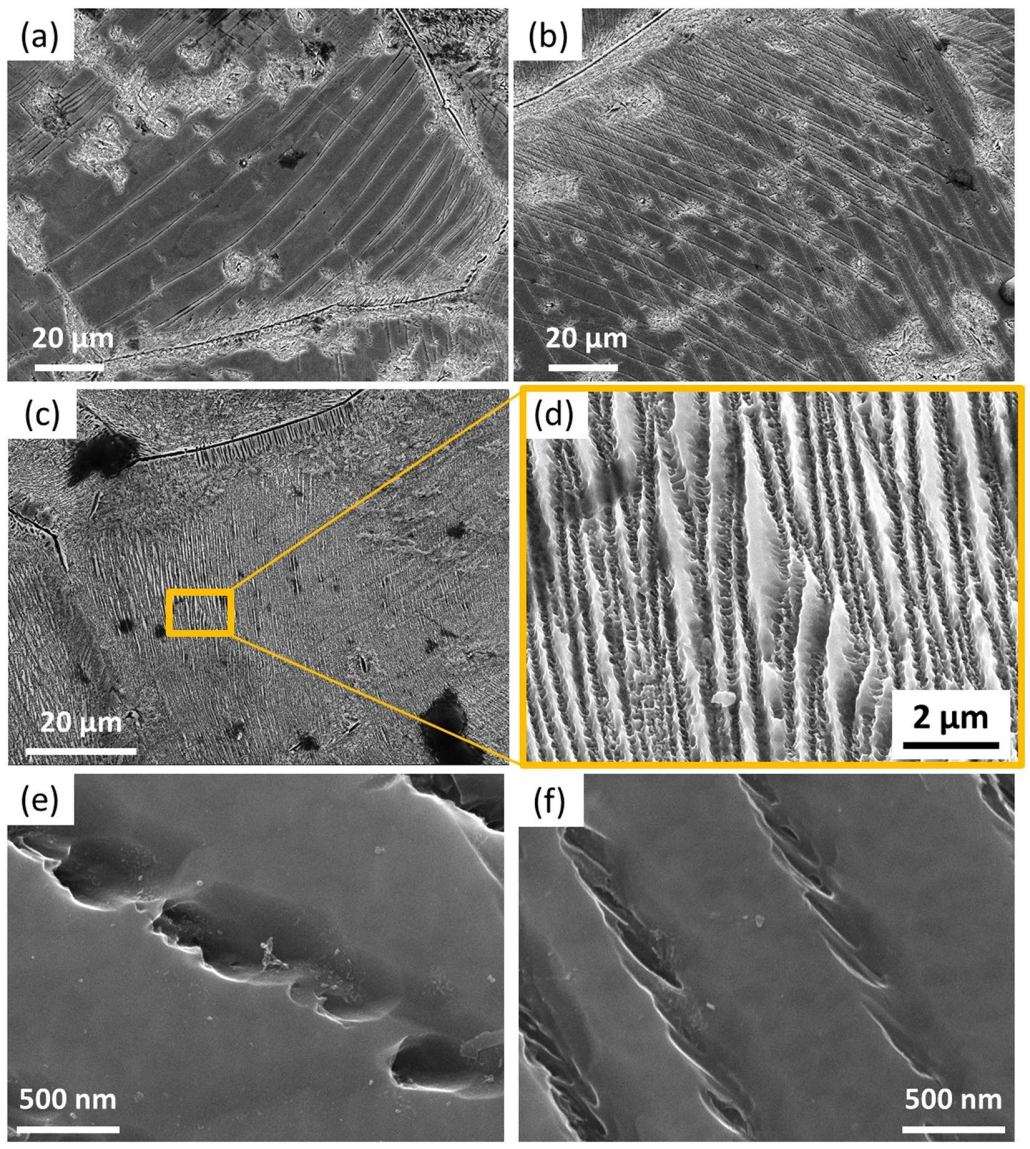
SEM secondary electron images of Ti6554 specimens deformed at 10000 s^−1^ and 873 K: (**a**–**c**) string-shaped morphologies; (**d**) enlarged view showing the high density of string-shaped morphologies from the area marked in (**c**); (**e**) aligned spherical morphologies; (**f**) aligned lath-shaped morphologies.

The string-shaped morphologies observed in the cylindrical samples after SHPB tests conducted at a strain rate of 10000 s^−1^ at 873 K are shown in Fig. 2. The compressive axis of SHPB tests is normal to the plane of the page. These morphologies are confined to individual β grains and the spacing between them varies. For example, the spacing between each string in Fig. 2a is over 10μm while in Fig. 2b it is markedly reduced. In Fig. 2c, a high density of strings was observed and the spacing between these strings obtained from a magnified image (Fig. 2d) is ~50–500 nm. These string-shaped morphologies were developed from the coalescence of preferentially aligned spherical or lath-shaped morphologies as shown in Fig. 2e and f, respectively. From SEM observations, the size of individual spherical morphology is ~300–500 nm while for individual lath-shaped morphology the length is ~300–700 nm and width ~40–100 nm. Moreover, two distinct orientations of the strings can be observed within some β grains as shown in Fig. 2b.

**Figure 3:**
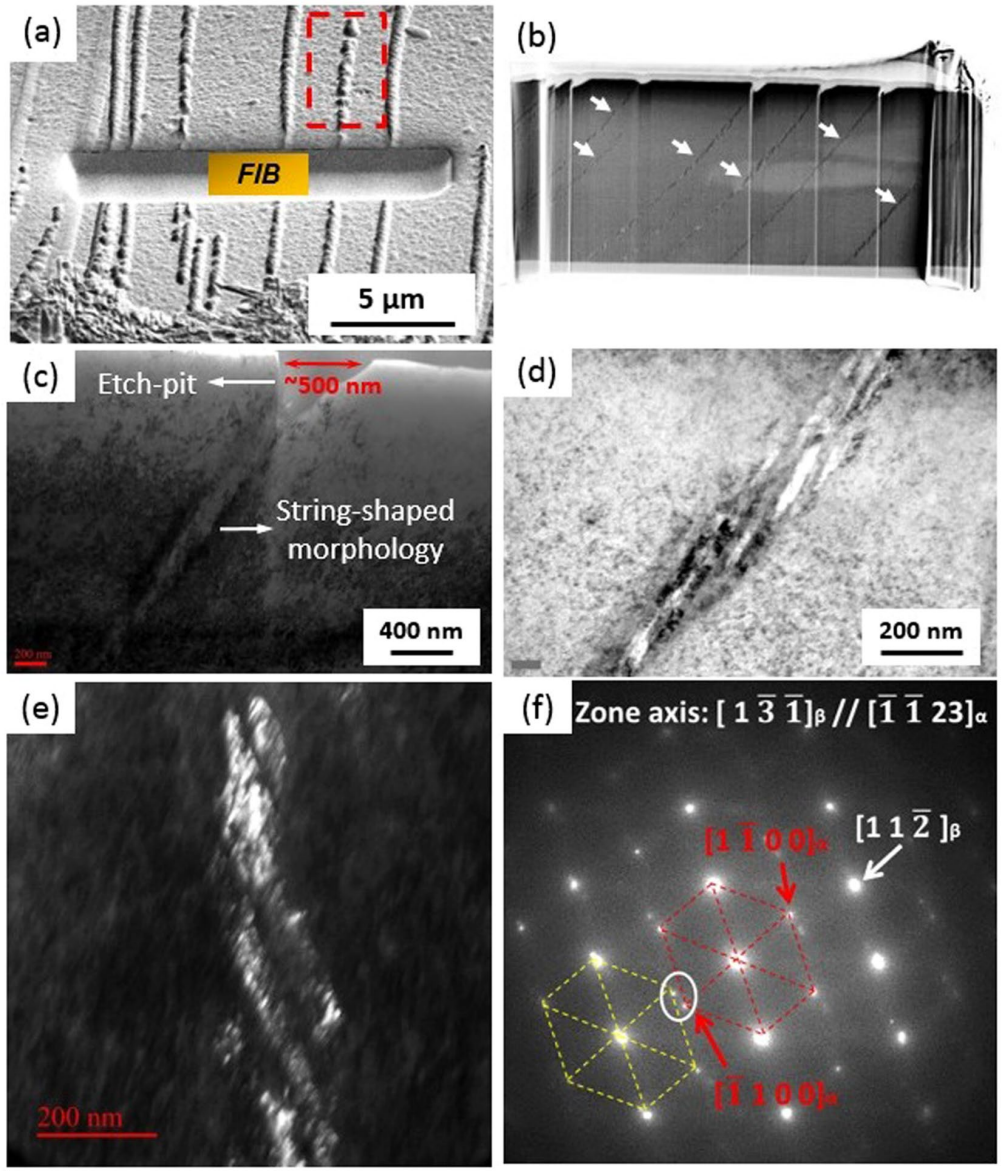
SEM secondary electron images of (**a**) the location where the FIB foil was lifted out; (**b**) the FIB specimen with string-shaped morphologies arrowed; TEM bright-field image of (**c**) a string-shaped morphology and its corresponding etch-pit and (**d**) string-shaped morphology at a higher magnification; (**e**) TEM dark-field image of α laths by selecting the diffraction spots circled in (**f**); (**f**) SAD pattern with beam direction paralleled to [$1\bar{3}\bar{1}$]_β_ corresponding to (**e**) (The yellow dashed lines indicate the reflections from double diffraction). The FIB sample was from the Ti6554 specimen deformed at 10000 s^−1^ and 873 K.

**Figure 4:**
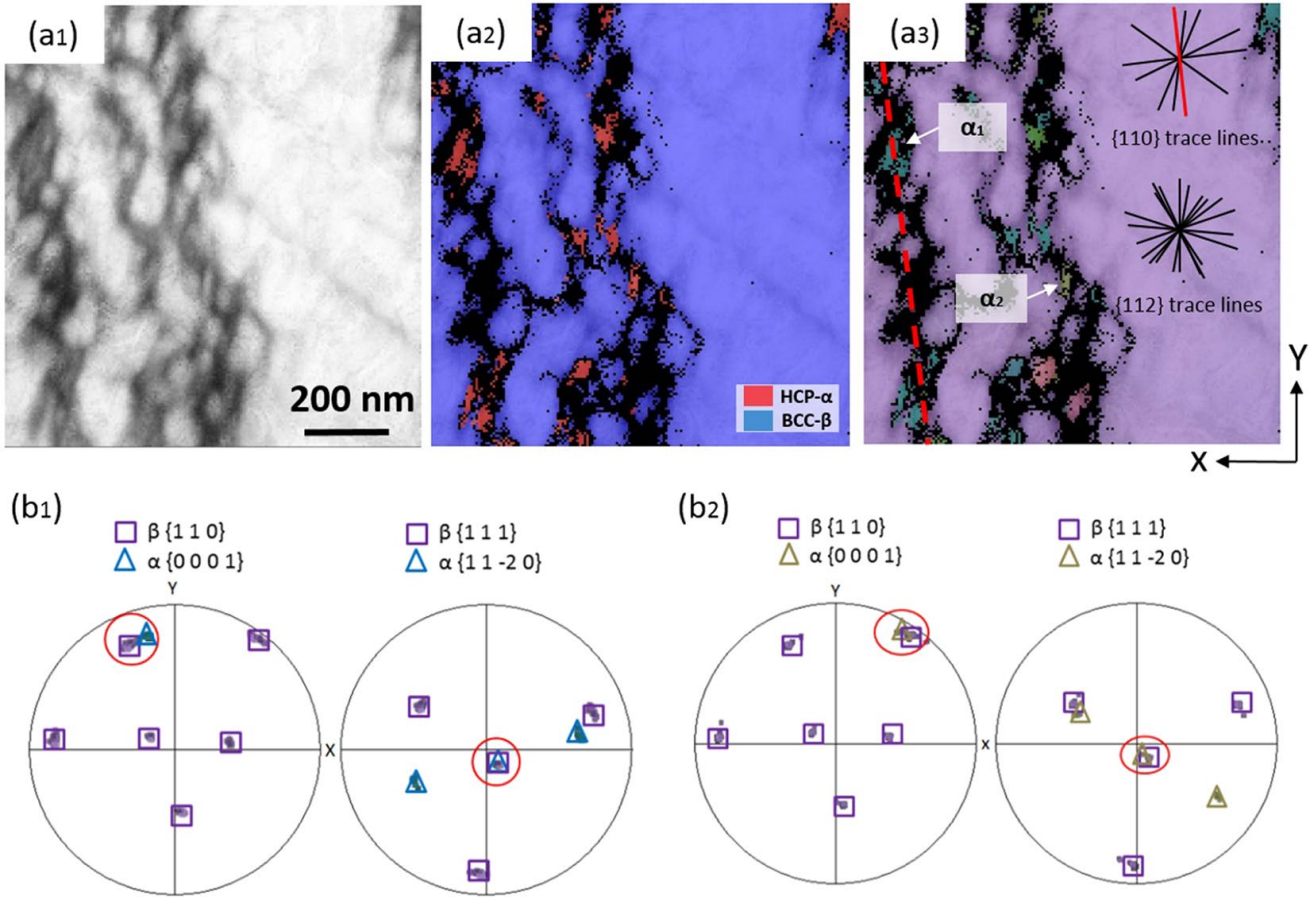
TKD results (step size = 6 nm) for string-shaped morphologies observed in Ti6554 specimens deformed at 10000 s^−1^ and 873 K: (**a**_**1**_) band contrast map, (**a**_**2**_) phase map and (**a**_**3**_) Euler map with trace lines of {110} and {112} slip planes plotted (the red coloured {110} trace line indicates a potential slip plane on which the coalescent α laths are aligned); (**b**_**1**_) and (**b**_**2**_): pole figures for α laths labelled in (**a**_**3**_) and their surrounding β matrix showing an approximate Burgers orientation relationship.

A FIB sample for TEM observations was prepared from the Ti6554 specimen deformed at 10000 s^−1^ and 873 K as shown in Fig. 3a. The foil is at approximately 90° with respect to the strings. These strings are formed by the coalescence of aligned spherical morphologies referring to the area marked by the dashed rectangle in Fig. 3a. Figure 3b is a SEM image of the FIB foil. It is found that the morphologies buried below each sphere are still in string shape (as indicated by white arrows). It indicates that the spherical morphology observed in Fig. 2e represents the region where a “string” come into contact with the section. Therefore, the term “etch-pit” is more suitable to describe it. As shown in Fig. 3c, the size of etch-pit is larger than the shorter axis of the string buried below it. A TEM image at a higher magnification (Fig. 3d) demonstrates that the strings shown in Fig. 3b consist of refined lath-like features with length of ~100–300 nm and width of ~20–40 nm. A TEM dark-field image of lath-shaped features and their corresponding selected area diffraction pattern (SADP) are shown in Fig. 3e and f, respectively. Figure 3e was formed by selecting the reflections circled in Fig. 3f. These lath-shaped features have been indexed to be α precipitates holding a specific variant with respect to β matrix. As the FIB sample is not able to be tilted to the 〈1 1 0〉 zone axis of β matrix due to limitations of the maximum tilting angle of the TEM equipment used, no image of edge-on α laths can be shown here^24^. To further understand the crystallographic orientation relationship between the aligned α laths and β matrix, TKD studies were also done on FIB samples. The band quality of the region surrounding the lath-shaped feature is significantly worse than that of the β matrix far away as shown in Fig. 4a_1_. In Fig. 4a_2_ some laths have been indexed to be α phase. However, some other laths are coloured black representing non-indexed area. This low indexing rate might be the consequence of the misfit strain existed between α lath and β matrix. α laths with different crystallographic orientations are labelled in Fig. 4a_3_ with their corresponding pole figures shown in Fig. 4b_1_ and b_2__,_ respectively. It can be found that both of the labelled α laths follow an approximate Burgers orientation relationship of {110}_β_ // {0001}_α_ and 〈$1\bar{1}1$〉_β_ // 〈11$\bar{2}$0〉_α_^25^. On the basis of SEM, TEM and TKD results, the appearance of the aligned α laths observed under SEM is very similar to that of α precipitates formed in the planer slip bands gliding on a specific plane as reported in refs^12,13,26^. As the typical slip systems activated in body-centred cubic (bcc) β-Ti phase are {110} 〈111〉 and {112} 〈111〉^14,27^, trace lines for {110} and {112} slip planes have been plotted in Fig. 4a_3_, following the method described in ref.^28^, based on the pole figures of {110} and {112} planes. The direction of preferential alignment of α laths is consistent with the surface trace of a {110} slip plane. It indirectly suggests that the aligned α laths may decorate the slip bands gliding on one of {110} planes.

**Figure 5:**
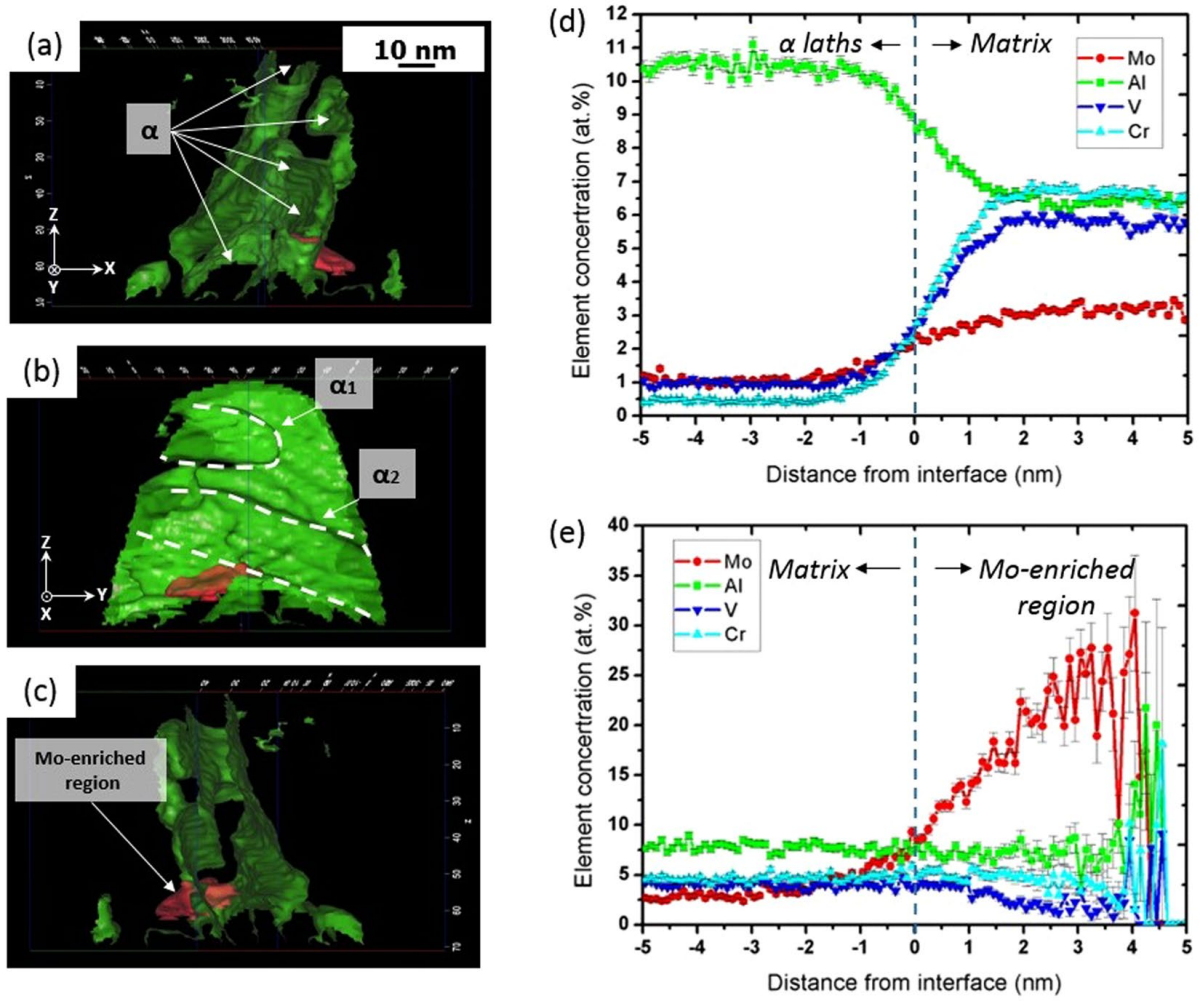
APT reconstruction of α laths from specimens deformed at 10000 s^−1^ and 873 K: (**a**)–(**c**) isosurfaces of Cr + V (6.53%) and Mo (8.68%) indicating α laths and Mo-enriched region; Al, Mo, V and Cr proxigrams obtained from: (**d**) the biggest V + Cr isosurface, (**e**) the Mo isosurface.

APT reconstruction in Fig. 5 provides an insight into 3D arrangement and composition of the aligned α laths. As Cr and V are typical β stabilisers, (Cr + V)-lean regions are designated to be α laths as delineated by green coloured iso-concentration surfaces (“isosurfaces” in short) of Cr + V = 6.53% (Fig. 5a–c). In addition, a Mo-enriched region enclosed by the isosurface in red (Mo = 8.68%) resides at the α/β interface (Fig. 5c). The interconnected (Cr + V)-lean zone is actually consisting of several α laths holding the same variant (Fig. 5a). Figure 5a,b (rotated ~90 degrees clockwise around the Z axis from Fig. 5a) are combined to show the arrangement that several α laths are aligned with their broad faces paralleled to each other (also referred to video in the supplementary material). The isosurface shown in Fig. 5b is actually an overlap of broad faces of aligned α laths and two of them are delineated by white dashed lines. The width of the broad face is estimated to be ~30–40 nm, which is of the same order as measured from TEM observations (Fig. 2). For an accurate composition analysis, proximity histograms (proxigram)^29^ of the V + Cr and Mo isosurfaces are shown in Fig. 5d,e, respectively. Figure 5d was plotted based on the biggest isosurface of Cr + V = 6.53%. It shows a significant partitioning in the β stabilizers: Cr, V and Mo; and also α stabilizer Al between α laths and β matrix: ~1.0% Mo, ~1.0% V, ~0.5% Cr and ~10.5% Al in α laths while ~3% Mo, ~5.5% V, ~6.5% Cr and 6.0% Al in β matrix. Figure 5e is plotted based on the Mo isosurfaces and therefore reveals an elemental partitioning between the Mo-enriched zone and the remainder of the APT needle. A strong Mo pile-up over 25% can be observed in the Mo-enriched region as shown in Fig. 5e. It is noted that an even stronger Mo enrichment of 40% at the α/β interface where two α sideplates intersected was previously reported by Nag *et al*.^30^ in the Ti–5Al–5Mo–5V–3Cr–0.5Fe alloy. It has been attributed to the overlapping diffusion fields from adjoining intersected α regions.

### Observation of adiabatic shear bands along with the string-shaped morphologies

**Figure 6:**
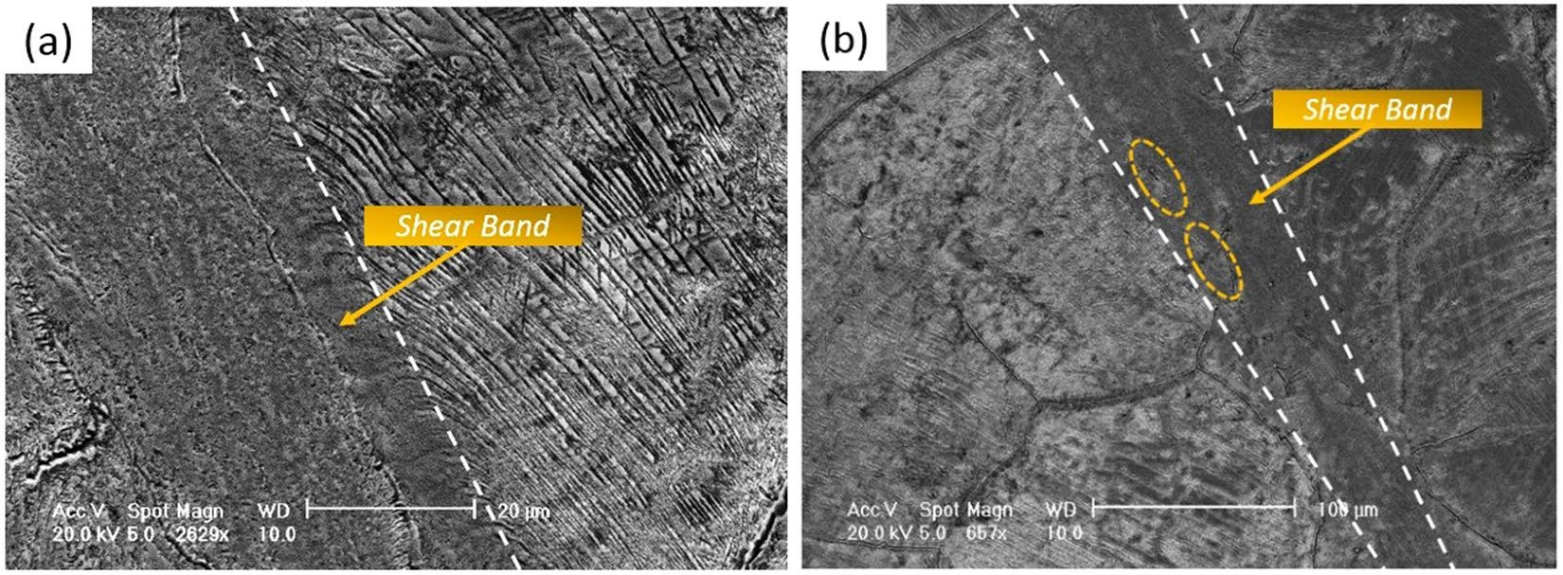
SEM secondary electron images showing adiabatic shear bands (boundaries have been delineated by white dashed lines) along with string-shaped morphologies: (**a**) in a Ti6554 specimen deformed at 4000 s^−1^ and 873 K: the string-shaped morphologies near the boundary were apparently curved toward the shearing direction; (**b**) in a Ti6554 specimen deformed at 10000 s^−1^ and 873 K: some residue of string-shaped morphologies within the shear band are marked.

As shown in Fig. 6, adiabatic shear bands (ASBs) can be observed along with the string-shaped morphologies. More detailed description of microstructures within the ASBs can be referred to ref.^31^. ASBs are the consequence of shear localization and always lead to catastrophic failure of materials during high-strain-rate deformations^32,33^. The formation of ABSs depends on deformation parameters (strain rate and temperature), strain level and microstructural characteristics. According to Table 1 ^21^, when the strain rate was 10000 s^−1^, ASBs were initiated in the specimens deformed at 873 K with a lower strain level (0.27) while absent in the specimens deformed at 293 K and 573 K with higher strain levels (>0.3). It goes against a general perspective that the tendency toward adiabatic shearing increases with decreasing temperatures and increasing straining^32,34^.

**Table 1:** Largest strain reached under different conditions in SHPB tests (“*” indicates that string-shaped morphologies and ASBs were observed).

	1000 s^−1^	4000 s^−1^	10000 s^−1^
293 K	~0.20	~0.22	~0.30
573 K	~0.20	~0.30	~0.35
873 K	~0.17	~0.37*	~0.27*
1173 K	~0.15	~0.25	/

## Discussion

**Figure 7:**
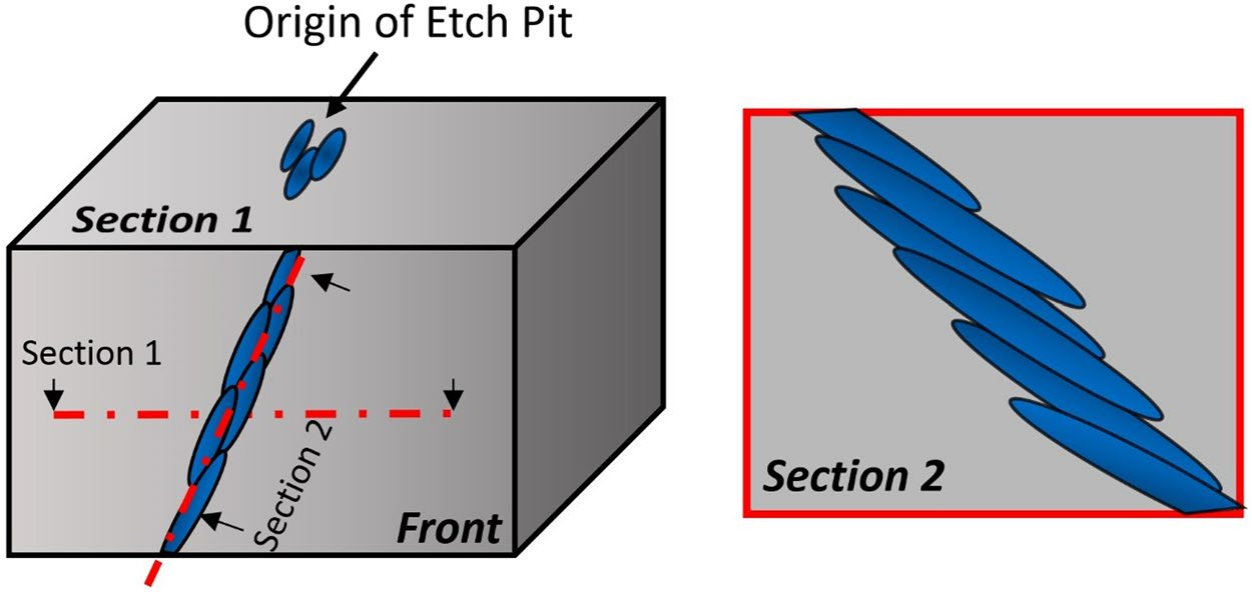
Schematic illustration showing the morphology and arrangement of the coalescent α laths. The front view and projected sectional views illustrate the 2D appearance of aligned α laths observed on different sections.

Schematics showing the morphology and spatial arrangement of the aligned α laths based on the TEM, TKD and APT results are plotted in Fig. 7. The 2D projected morphology of the coalescent α laths observed in the etched samples under SEM observations depends on the metallographic section. When the spacing between individual strings on the section 2 is smaller than the size of etch-pit, the corresponding etch-pits observed on the section 1 will also coalesce to form very similar string-shaped morphologies as shown in Figs 2e and 3a.

With regard to the formation mechanism of the string-shaped morphologies, all the experimental results presented so far strongly suggest that dislocation structures induced by high-strain-rate deformations play a key role in the nucleation of the aligned α laths, even though the image of initial dislocation structures has not been captured: (i) the appearance of the string-shaped morphologies consisting of aligned α laths is very similar to that of slip bands observed in the deformed β titanium alloys as reported in the previous publications^12,35,36^; (ii) the clear variant-selected behaviour of the aligned α laths is probably ascribed to the influence of strain field around dislocation structures since a parallelism is always retained between the Burgers vector of dislocation and the direction of the maximum misfit between α precipitates and β matrix (sub-grain boundary is an alternative explanation but no similar appearance of sub-grain boundaries has ever been reported)^10,12,14,17,37^; (iii) the existence of dislocation structures will contribute to the accelerated kinetics of nucleation and elemental partitioning of the aligned α precipitates, otherwise the diffusion-controlled α precipitation is unlikely to occur in such a short timeframe^38–41^; (iv) there are many examples in the literatures for the observations of α precipitates growing on the slip bands in the pre-strained β titanium alloys after ageing heat treatment^10,13,16,26,42^. Another indirect supportive point is that the string-shaped morphologies were absent in the specimens high-strain-rate deformed at 1173 K. It is speculated that the dislocation structures introduced by deformations were annihilated by strong recrystallization behaviour occurring at 1173 K.

It is worth noting that a significant temperature dependence is associated with this phase transformation as the features were only observed in the specimens deformed at 873 K referring to Table 1. Due to the very limited duration of high-strain-rate deformations (20–50 μs), the entire process should be regarded as non-isothermal and post-deformation air cooling process should play a key role in solute diffusion. The absence of coalescent α laths after deformations at 573 K and 293 K is due to the fact that the very limited diffusion rate of solutes at low temperatures retards the nucleation and growth of α phase. The string-shaped morphologies, however, were also absent in the specimens after high-strain-rate deformations at 1173 K. As mentioned earlier, the dislocation structures introduced by deformations were annihilated by the dynamic recrystallization occurring at 1173 K^21^. Thus, the lack of heterogeneous nucleation sites impeded the occurrence of the string-shaped morphologies. Unfortunately, the dependence of this α precipitation behaviour on strain rate cannot be clarified based on the current experimental data as the final strains reached at 873 K are in a relatively wide variation with different strain rates (Table 1). Therefore, more systematic and strictly strain-controlled experiments would be required to separate the influence of strain and strain rate.

Since solutes can keep diffusing before samples being air-cooled to the temperatures where diffusion rate becomes very limited, the growth and elemental partitioning of the aligned α laths will keep proceeding during air-cooling process after high-strain-rate deformations end. Moreover, the timeframe of air-cooling process following deformations (at second level) is much longer than high-strain-rate deformations (at micro-second level). Therefore, it remains unclear that whether the aligned α laths formed during high-strain-rate deformations or after deformations by post-deformation ageing effects and the evolution process of the aligned α precipitates will be difficult to observe. Despite of this, some deductions can be made based on the abnormal initiation of ASBs during deformations at 873 K with a relatively low strain level. It is noted that microstructural characteristics affect the susceptibility of alloys to adiabatic shearing^43^. According to the perturbation theory proposed by Duffy *et al*.^44^, the increment in volume fractions of second-phase particles, precipitates or other inhomogeneity will trigger the adiabatic shearing at a lower critical strain. Previous high-strain-rate tests on steels conducted by Odeshi *et al*.^45–47^ also support this theory. Similarly, the string-shaped morphologies were probably formed during high-strain-rate deformations and enhanced the inhomogeneity of microstructures, thereby increasing the susceptibility of the alloy to adiabatic shearing. On the other hand, the initiation of ASBs may also contribute to the formation of more aligned α laths near the ASBs (Fig. 6a) since the severe shear deformations occurring near the boundary of ASBs will supply substantial amount of dislocation structures for the nucleation of α precipitates.

In a summary, the string-shaped morphologies observed in the metastable β-Ti6554 alloy deformed at high strain rates and elevated temperatures have been clarified to consist of aligned lath-shaped α precipitates. 2D morphological pattern of the coalescent α laths observed in the etched samples by SEM depends on the metallographic section. The appearance similarity between slip bands and string-shaped morphologies under SEM observations, strong variant-selected behaviour and accelerated kinetics in the elemental partitioning of α laths all suggest that dislocation structures are most likely the nucleation sites for the aligned α laths. In addition, an appropriate testing temperature, which can ensure a relatively high diffusion rate of solutes without inducing strong recovery of dislocation structures, is necessary for the occurrence of the features.

## Methods

Hot rolled cylindrical rods of the Ti6554 alloy were solution treated at 1100 K for 1 h in a protective argon atmosphere and then air-cooled. Aging treatments were conducted on the solution treated Ti6554 rods at 833 K for 8 h in the ambient atmosphere and samples were again air-cooled. The chemical composition of as-received materials is shown in Table 2 and the details of SHPB tests have been reported elsewhere^21^. For the experiments conducted at elevated temperatures, specimens were heated by an *in-situ* induction coil and temperature was regulated by a thermocouple not in contact with the specimens. The heating rate cannot be controlled accurately and it took around ~600 s to heat the specimens from room temperature to 573 K and ~1200 s to 1173 K.

**Table 2:** Chemical composition of the Ti-6Cr-5Mo-5V-4Al alloy.

Chemical Component (wt. %)					
Cr	Mo	V	Al	O	Ti
6.05	4.95	5.09	4.20	0.19	Bal.

The specimens for microstructural observation and X-ray diffraction (XRD) test were wet ground using silicon carbide papers, mechanically polished and ultrasonically cleaned. XRD was conducted on a D8 Advance X-ray diffractometer equipped with a graphite monochromators and a Ni-filtered Cu K_α_ source. The specimens for optical and SEM observations were etched using Kroll’s reagent (2% hydrofluoric acid, 6% nitric acid and 92% distilled water). SEM was performed on a JEOL JSM-7001F instrument. A FEI-SCIOS Focused Ion Beam (FIB) was used to prepare site-specific specimens for TEM and TKD observations. TEM investigations were performed using JEOL2100 and TECNAI20 microscopes operated at 200 kV while TKD investigations were performed using Zeiss Ultra-Plus FEG SEM, equipped with an Oxford Channel 5 EBSD system.

The preparation method applied for needle-shaped APT samples was the same as described in ref.^48^. APT experiments were conducted in a CAMECA LEAP 4000× instrument. Data acquisition was carried out in laser pulsing mode at a specimen temperature of 48.5 K with a target evaporation rate of 5 ions per 1000 pulses, a detection rate of 1%, a pulse rate of 250 kHz, laser energy of 50 pJ and a laser spot size of approximately 2 mm. The wavelength of the UV laser was 355 nm. The APT data were reconstructed and analyzed using commercial IVAS 3.6.8 software with a detection efficiency of 0.57, k factor of 3.3 and image compression factor of 1.65.

The datasets generated during and/or analysed during the current study are available from the corresponding author on reasonable request.

## Electronic supplementary material

Supplementary Information
